# Encoding force modulation in two electrotactile feedback parameters strengthens sensory integration according to maximum likelihood estimation

**DOI:** 10.1038/s41598-023-38753-y

**Published:** 2023-08-01

**Authors:** Shima Gholinezhad, Dario Farina, Strahinja Dosen, Jakob Dideriksen

**Affiliations:** 1 Department of Health Science and Technology, Aalborg University, Aalborg, Denmark; 2 Department of Bioengineering, Faculty of Engineering, Imperial College London, London, SW7 2AZ UK

**Keywords:** Biomedical engineering, Sensory processing

## Abstract

Bidirectional human–machine interfaces involve commands from the central nervous system to an external device and feedback characterizing device state. Such feedback may be elicited by electrical stimulation of somatosensory nerves, where a task-relevant variable is encoded in stimulation amplitude or frequency. Recently, concurrent modulation in amplitude and frequency (multimodal encoding) was proposed. We hypothesized that feedback with multimodal encoding may effectively be processed by the central nervous system as two independent inputs encoded in amplitude and frequency, respectively, thereby increasing state estimate quality in accordance with maximum-likelihood estimation. Using an adaptation paradigm, we tested this hypothesis during a grasp force matching task where subjects received electrotactile feedback encoding instantaneous force in amplitude, frequency, or both, in addition to their natural force feedback. The results showed that adaptations in grasp force with multimodal encoding could be accurately predicted as the integration of three independent inputs according to maximum-likelihood estimation: amplitude modulated electrotactile feedback, frequency modulated electrotactile feedback, and natural force feedback (r^2^ = 0.73). These findings show that multimodal electrotactile feedback carries an intrinsic advantage for state estimation accuracy with respect to single-variable modulation and suggest that this scheme should be the preferred strategy for bidirectional human–machine interfaces with electrotactile feedback.

## Introduction

Human–machine interfaces are often unidirectional: a physiological signal is recorded and used to control an external device. In many applications of these technologies, however, a bidirectional interface may increase performance. For example, mechatronic upper-limb prostheses can be controlled with electromyographic signals^1^, but users often over-rely on visual cues, thereby minimizing the amount of visual attention that can be directed elsewhere even during simple manual tasks^2^. For this reason, restoring some level of somatosensory feedback may improve the utility and ease of use of the system^3,4^. Fundamentally, delivering such feedback implies evoking a sequence of action potentials associated with a task-relevant variable in a set of somatosensory nerves^5^. In the context of prostheses, the action potentials that encode the sensory information measured from the prosthesis (e.g., grasp force) are typically evoked by electrical stimulation or vibration, and their generation (number and timing), controlled by either amplitude modulation (AM) or frequency modulation (FM)^6–8^.

Recently, several studies have proposed encoding the feedback variable using concurrent modulation in both frequency and amplitude, for invasive^9,10^ and non-invasive^11^ peripheral nerve stimulation. Hereafter, we refer to this strategy as multimodal encoding. For example, using intraneural stimulation in two amputee subjects, Valle and colleagues found such multimodal encoding to be superior to AM and FM with regards to performance in functional tasks and subjective rating^9^. The rationale of using multimodal modulation was that it is biomimetic since AM controls the number of activated nerve fibers (recruitment) and FM controls the rate of action potentials evoked in these fibers (rate coding). Indeed, in the encoding of stimulus intensity by groups of natural sensory receptors, including cutaneous receptors^12^, Golgi tendon organs^13^, and muscle spindles^14^, the recruitment and rate coding are intrinsically linked: An increase in stimulus intensity implies an increase in the discharge rate of action potentials in the active fibers and recruitment of additional fibers (until full recruitment of all receptors). This apparent similarity between natural encoding and artificial multimodal stimulation suggests that the latter may elicit sensations that resemble natural feedback, making it more intuitive for the brain to interpret compared to sensations evoked in paradigms relying on unimodal modulation (AM or FM alone).

Another factor that may explain or contribute to the promising results obtained with the multimodal approach is that concurrent AM and FM provides more sensory information which allows the brain to estimate the feedback variable more accurately. In the process of estimating the state of a limb, it is well established that the nervous system integrates information from all relevant sensory sources according to maximum likelihood estimation (MLE) to obtain an estimate that is more accurate than what could be achieved with each of the sources alone^15,16^. For example, localization of a stimulation point on the skin is more accurate when activating both tactile and nociceptive receptors, than with individual activation of the two classes of receptors^17^. Concurrent modulation of stimulation amplitude and frequency can evoke two distinct percepts since electrical stimulation generates action potentials in all recruited nerves synchronously. Consequently, changing frequencies determines the stimulation quality (from discrete tapping to vibration with increasing frequency^18^) while the stimulation amplitude sets the intensity of this sensation. Moreover, the degree of interaction between these two evoked percepts is relatively small^19,20^. In practice, this implies that during AM, a subject has an accurate perception of the frequency by which the electrical pulses are delivered and vice versa for FM^21^.

In this study, we assessed performance in a grasping force matching task, where a total of 15 subjects (including one amputee) received supplementary feedback. This feedback was delivered by electrotactile stimulation reflecting the instantaneous force with multimodal, AM, or FM encoding. Using an adaptation paradigm^22,23^, the mapping between force and electrotactile stimulation was perturbed in a subset of trials. Importantly, this perturbation was unnoticeable for the subjects, forcing them to subconsciously estimate the force as a compromise (weighted average) between the natural and electrotactile force feedback. The result of this compromise (i.e., the force magnitude produced when attempting to match the target force) in biased trials revealed the relative weight assigned to each feedback in the force estimation process according to MLE: If the matching force was affected only to a small degree by the perturbation, the natural feedback had the largest influence on the estimated force, and thus the highest relative weight, and vice versa. We hypothesized that multimodal encoding would have the highest relative weight and thus enable the most accurate force matching ability. Moreover, we hypothesized that this advantage could be explained by the CNS effectively perceiving the stimulation as two sensory inputs describing the same feedback variable through the electrotactile stimulation: one encoded in amplitude and one in frequency. According to MLE, the force perception obtained by integrating natural force feedback and two, as opposed to one, electrotactile feedback signals would provide a more reliable estimate of the force produced. We tested this hypothesis by predicting the behavior (relative weight of electrotactile feedback and matching force variance) when receiving multimodal encoded feedback based on the results from the AM and FM conditions and comparing the outcome with experimentally observations with multimodal encoding. Recently, using this experimental technique, we demonstrated that electrotactile and natural force feedback are indeed integrated in accordance with the MLE^24^. With this study, we investigate if the same principle extends to multimodal modulation schemes.

## Results

### Psychometric assessment and subjective ratings

Before starting the adaptation paradigm, thresholds for stimulation sensation and discomfort (ST, DT) to the stimulation were identified to identify the appropriate stimulation settings. On average, these thresholds were 11.78 ± 2.19 and 32.35 ± 4.14 mA in amplitude modulation, 11.64 ± 2.34 and 34.35 ± 4.30 mA in frequency modulation, and 11.28 ± 2.84 and 33.5 ± 4.70 mA in multimodal modulation. The statistical analysis showed no significant differences among the three modulations for ST and DT. The ST and DT of the amputee participant were within the range of the able-bodied subjects (14 and 30 mA respectively, in multimodal modulation).

Next, the Just Noticeable Difference (JND) to stimulation was identified to ensure that the perturbation in the force matching task was unnoticeable for the subject. On average, the JND in multimodal modulation (JND_multi_ ≃8.98 ± 5.41%) was significantly lower ($p = .001$), compared to amplitude (JND_AM_ ≃ 17.11 ± 8.39%) and frequency modulation (JND_FM ≃_19.29 ± 8.64%). Pairwise comparisons indicated that discrimination ability was significantly higher when modulating the two parameters compared to modulating amplitude only (JND_multi_ vs. JND_AM_, $p = 0.002$) or frequency only (JND_multi_ vs. JND_FM_, $p = 0.003$). The JND for amputee subject was 7.00% in the multimodal modulation condition. During the primary experimental task, none of the subjects noticed that the mismatch between the electrotactile stimulation and natural feedback was introduced.

After the force matching task, the subjects rated the multimodal modulation as being most efficient with respect to how well the stimulation signaled the grip force as well as being most comfortable. Specifically, the efficiency scores were 5.6 ± 0.7 with AM, 5.9 ± 0.9 with FM and 7.4 ± 0.9 with multimodal, while the ratings of pleasantness were 5.21 ± 1.31, 6.71 ± 1.06 and 7.00 ± 1.17 for AM, FM and multimodal respectively. The ratings of the amputee subject corresponded to those of the able-bodied subjects (efficiency: 7.00; pleasantness: 7.00). The Friedman test revealed a significant difference between the three conditions in terms of efficiency ($p<0.001$) and pleasantness ($p = 0.005$). Applying the pairwise comparisons using Wilcoxon rank sum test, we found that the multimodal condition has been reported as a more efficient feedback scheme compared to both single-feature encodings: (multimodal vs. AM: $p< 0.001$; multimodal vs. FM: $p< 0.001$). Also, we found that subjects rated the multimodal and frequency modulated stimulation as significantly more pleasant compared to the amplitude condition (AM vs. FM: $p= 0.008$; AM vs. multimodal: $p= 0.005$).

### Performance in force matching task and relative feedback weights

**Figure 1:**
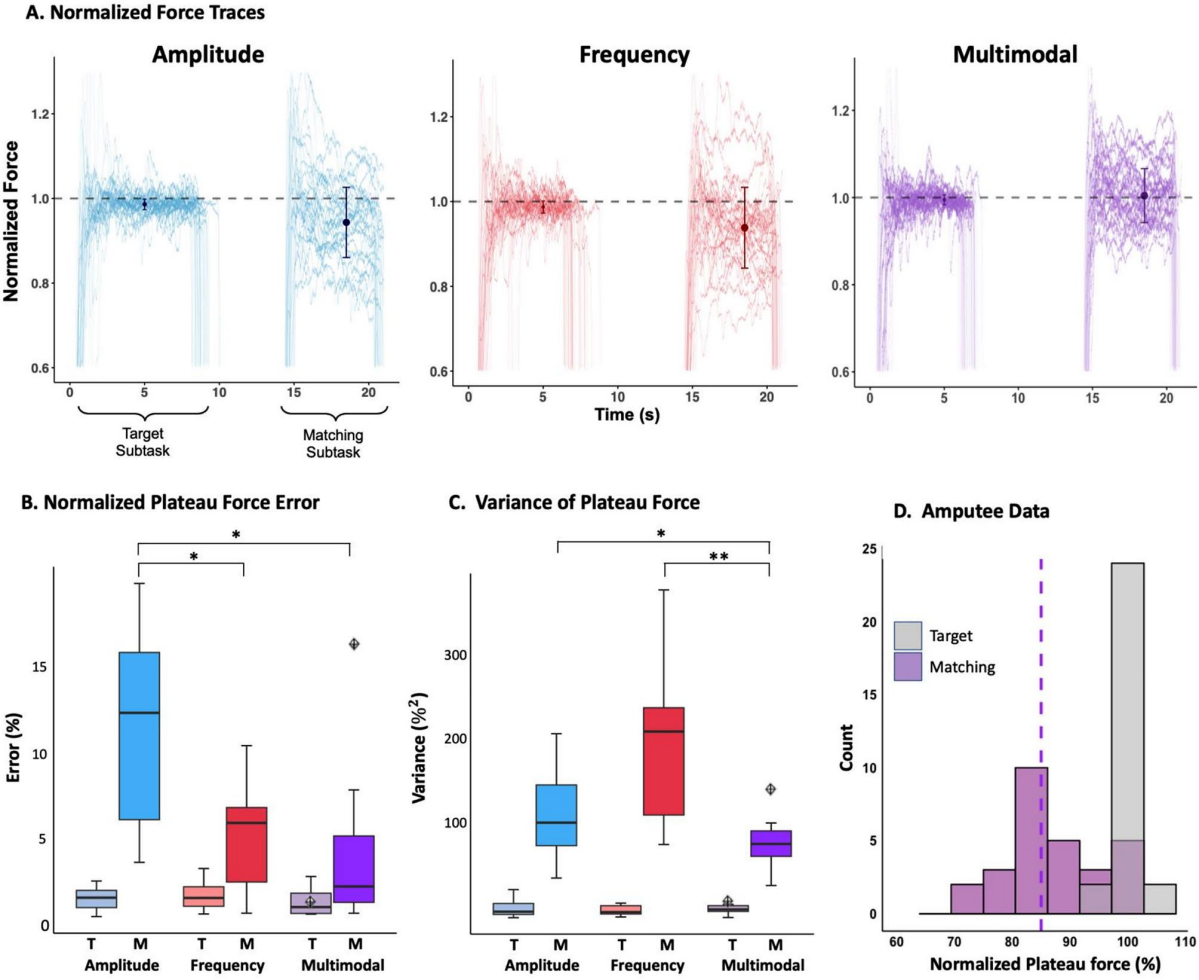
(**A**) Shows representative force traces normalized to the respective target forces during baseline condition for amplitude modulation (blue), frequency modulation (red), and multimodal modulation (purple). Each force trace consists of two contractions, representing target and matching forces. The horizontal grey dashed line represents the target force (normalized force = 1), and the error bar indicates the average ± standard deviation of normalized plateau force for each encoding scheme. (**B**,**C**) Show plateau force error and variance of normalized plateau force during the baseline condition for target (T) and matching (M) subtasks across all subjects. The diamond shape represents the amputee subject data in the multimodal condition. Distribution of normalized matching task plateau forces across the baseline condition for the amputee subject is shown in (**D**). The purple dashed line represents the mean of the matching distribution. Statistical significance is denoted by *p ≤ 0.05 or **p ≤ 0.001.

In each trial of the grasp force matching task, the subjects first generated the target force (randomly selected in the range 15–30% of maximum voluntary contraction level; MVC) with visual force feedback on a screen as well as natural and electrotactile force feedback. After a 5-s pause, the visual feedback was removed, and the subject was asked to recreate the target force by relying only on natural feedback and electrotactile stimulation (“matching” subtask). Figure 1A shows the normalized force traces generated by one representative subject in the baseline conditions for the three encoding schemes. In the target subtask, the generated force traces (approx. the first 7 s) matched the target force (the dashed line) accurately and precisely in all three conditions due to the presence of the visual feedback. However, in the absence of vision (matching subtask), the error and variance of the generated plateau force were noticeably higher. The error bars shown for each individual modulation represent the average and standard deviation of the normalized plateau force values across 30 repetitions. Comparing the three conditions, the plateau forces (i.e., average matching trial force) in multimodal modulation were produced with less variability and error reflecting higher accuracy and precision.

All subjects generated more accurate force signals (Fig. 1B) with reduced variability (Fig. 1C), when receiving visual feedback during the target subtask. Furthermore, the multimodal modulation scheme showed the lowest error (Fig. 1B) and variance (Fig. 1C) compared to the unimodal modulation settings. Applying repeated measure ANOVAs, we found significant differences across the three encoding schemes for error ($$p=0.004$$) as well as for the variance ($$p< .001$$). The pairwise comparison revealed that with the multimodal modulation, the average plateau force in the matching task was more accurate (less error) compared to amplitude modulation ($$p = .012$$) and less variable compared to both amplitude and frequency modulation (multimodal vs. amplitude: $$p=0.031$$ multimodal vs. frequency: $$p=0.003$$). Moreover, the comparison between the amplitude and frequency modulations revealed significant difference between the two groups in error ($$p=0.03)$$, but not in variance (Fig. 1B,C). In Fig. 1D, the distribution of normalized plateau force generated by the amputee subject for the baseline setting is depicted. As expected, in the target subtask, the amputee subject produced more accurate and less variable force. When examining the matching subtask, we observed a variance of 139%^2^, which aligns with the findings from the able-bodied subjects. However, there was a general tendency for the amputee subject to underestimate force in the matching task (83.68 ± 11.80% of target force), resulting in lower accuracy (Fig. 1B).

**Figure 2:**
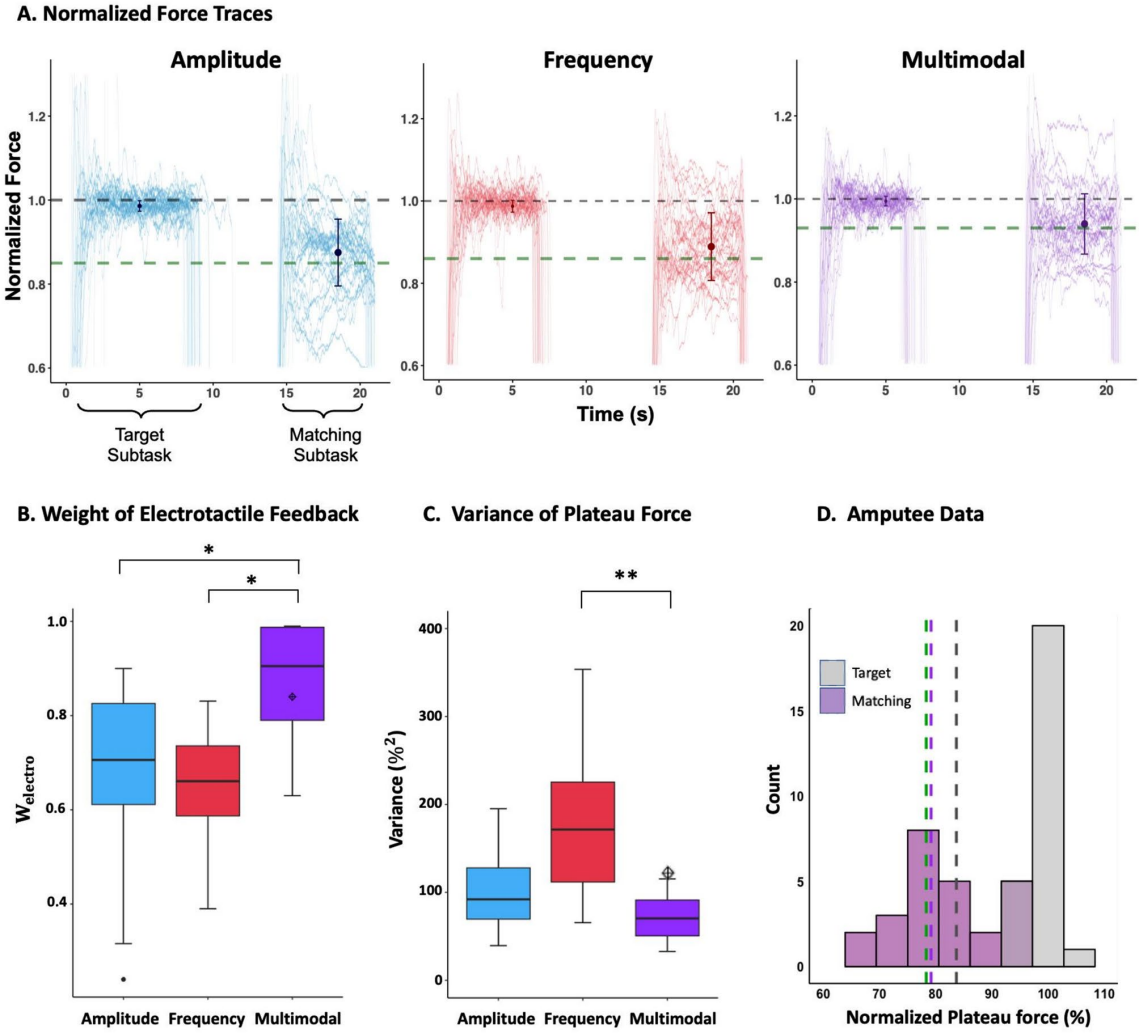
(**A**) Shows representative normalized force traces generated by the same subject as in Fig. 1A during the biased condition for amplitude modulation (blue), frequency modulation (red), and multimodal modulation (purple). The grey dashed line represents the target force (normalized force = 1), and the green dashed line indicates the force magnitude required to achieve the same electrotactile stimulation intensity (amplitude, frequency, or both) in the target matching task as received in the target task. The error bar indicates the average ± standard deviation of the normalized plateau force for each encoding scheme. (**B**,**C**) Shows the weight of electrotactile feedback and variance of normalized plateau force extracted from the biased condition across all subjects. The diamond shape represents the amputee subject data in the multimodal condition. Distribution of matching task plateau forces for the amputee subject across the biased condition is shown in (D). The vertical grey dashed line indicates the average of the plateau force distribution in non-biased trials, and the green dashed line indicates normalized plateau force required to achieve the same electrotactile stimulation intensity in the target matching task as received in the target task, adjusted to compensate for systematic deviations from the reproduction of the target force in the baseline trials (see Eq. 4), The purple line represents the mean of the matching distribution. Statistical significance is denoted by *p ≤ 0.05 or **p ≤ 0.001.

Figure 2A illustrates normalized force traces from a representative subject (the same subject as shown in Fig. 1A) the biased trials. By comparing Figs. 1A and 2A, it is evident that the subject generated lower matching task plateau forces in the biased condition, reflecting a compromise between recreating the sensations from natural force feedback and electrotactile feedback experienced in the target task. By analyzing the adaptation to the perturbations in the biased condition trials, the empirical relative weight assigned to electrotactile feedback was identified according to MLE. This weight was significantly higher in multimodal 0.86 ± 0.02 condition compared to AM (0.67 ± 0.061; $p=0.005$) and FM (0.65 ± 0.034 $;p=0.001$) (Fig. 2B).

As in the baseline condition, the biased trials with multimodal modulation showed lower variance across the trials. (Fig. 2C). Statistical analysis using repeated measures ANOVA revealed significant differences in variance across the three encoding schemes (p < 0.001), primarily driven by the significant difference between multimodal modulation and frequency modulation. No significant difference in variance was found between multimodal modulation and amplitude modulation (Fig. 2C). The estimated relative weight of the electrotactile feedback in the amputee participant (0.84) and the observed plateau force variance (103%^2^) fell within the range of values observed in able-bodied subjects. In Fig. 2D, the performance of the amputee subject in the biased setting is displayed for both the target and matching subtasks. Consistent with the baseline condition, a significantly higher variance and error in the matching force was found compared to the target force. Additionally, a consistent tendency to underestimate the matching force was observed. However, in response to the bias, the matching task plateau force was reduced to a degree where the average value (79.17 ± 10.15; Fig. 2D) was close to the level required for the electrotactile stimulation intensities to be similar across the target and matching tasks.

### Prediction of multimodal encoding weight

**Figure 3:**
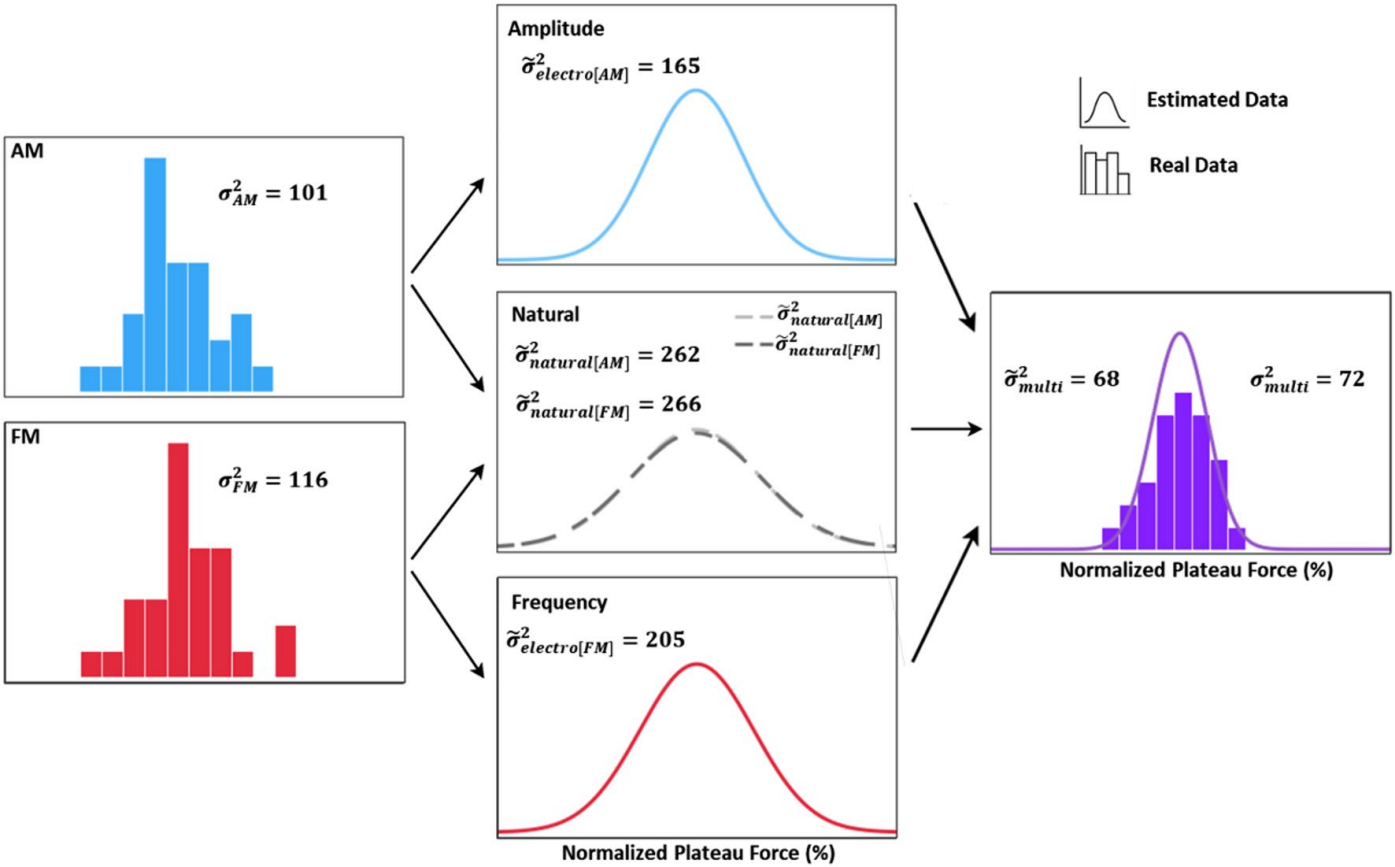
A representative illustration of the prediction of the variance of the plateau force in multimodal modulation condition based on the MLE framework using on the data collected in one subject. First, the variances of the experimentally observed distributions of matching task plateau forces in AM and FM are calculated. Based on the relative weight of the electrotactile feedback (${W}_{electro}$) from these conditions and Eqs. (10) and (11), we estimate the variance of each sensory input (electrotactile feedback: AM and FM; and natural feedback). By combining these three sensory inputs, we predict the variance for the feedback with multimodal modulation using Eq. (12) and compare it to the experimentally observed variance for the multimodal condition. Here, the variance of the natural feedback was set to the average of the two estimates obtained from AM and FM, respectively. The estimated variances are denoted in the figure as ${\widetilde{\sigma } }^{2}$ while the experimentally measured values are indicated using ${\sigma }^{2}$ and both are expressed in the units of normalized plateau force.

The main objective of the data analysis was to test the hypothesis that the performance in the force matching task with multimodal encoding could be predicted from the performance with AM and FM according to MLE. Specifically, this hypothesis implied that effectively three independent inputs were available for the estimation of grasp force: Natural feedback, as well as electrotactile feedback through AM and FM. Each of these inputs will reflect the grasp force with a certain variance. By estimating and integrating the variance of each independent input (obtained in the AM and FM conditions), the variance in trials with multimodal encoding could be predicted and compared to the variance obtained experimentally from those trials. This procedure is illustrated in Fig. 3 using data from one representative subject. In this case, the variance of the multimodal condition was accurately predicted from the AM and FM conditions.

**Figure 4:**
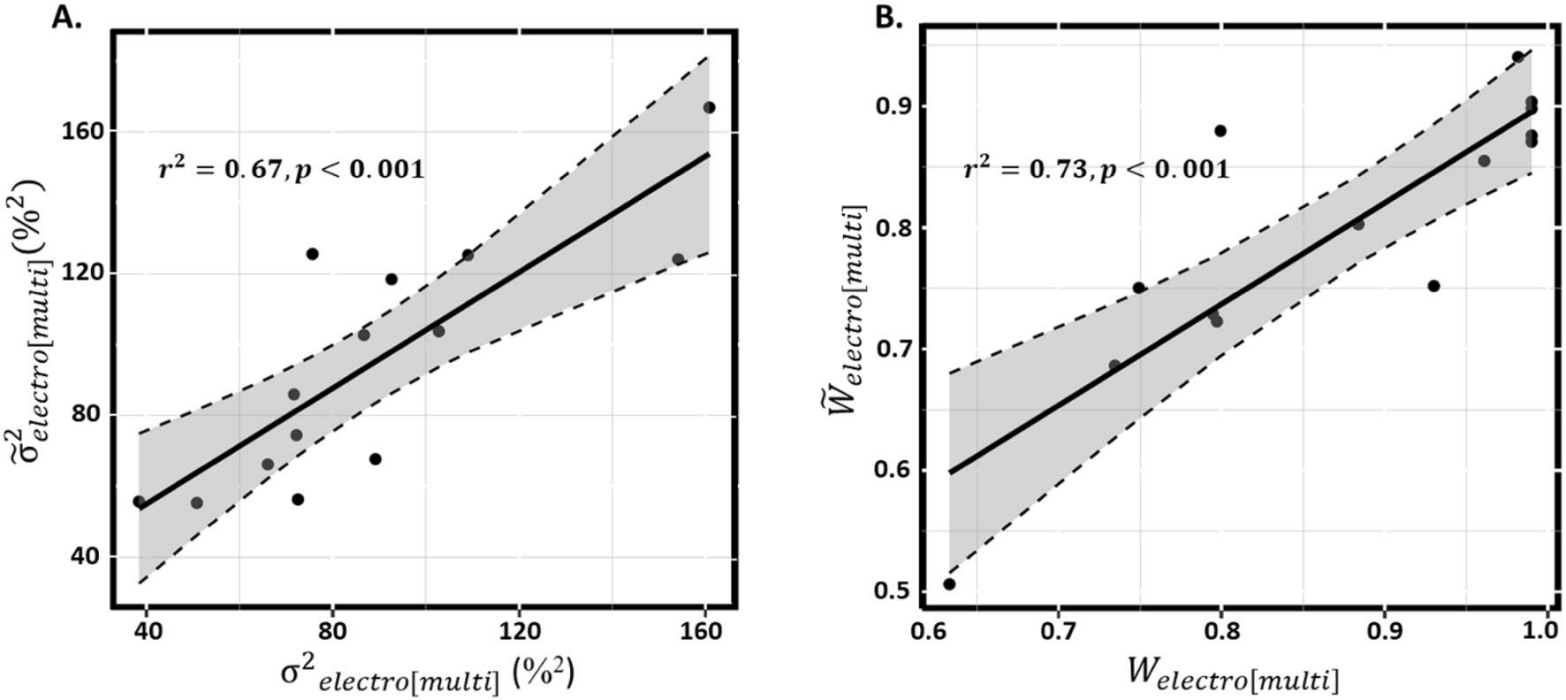
Scatter plot showing the relation between observed and predicted values (**A**) for variance of plateau force ($$y=1.02x+2.93)$$ and (**B**) the weight assigned to electrotactile feedback ($$y=0.83x+0.07$$) for multimodal modulation condition. The solid line indicates the best linear fit, and the dashed lines indicate the 95% confidence intervals for the regression lines.

Comparing the predicted and measured variance as well as the weight of electrotactile feedback in the multimodal modulation across all subjects (Fig. 4), we observed significant correlations for both parameters (variance: ${r}^{2} = 0 67, p < 0.001$. ${W}_{electro}$: ${r}^{2} = 0.73, p < 0.001$), indicating that the performance with multimodal modulation can be modelled accurately by the MLE-integration of AM and FM as two separate inputs.

## Discussion

We evaluated the subject’s performance in a grasp force matching task using supplementary electrotactile feedback with multimodal encoding (i.e., concurrent AM and FM). We hypothesized that the central nervous system perceives concurrent AM and FM in the same feedback signal as two separate sources of task-relevant information, which would allow a more accurate force estimate and thus a better task performance with multimodal compared with unimodal encoding. The results supported this hypothesis, as the weight of the electrotactile feedback (relative to natural grasp force feedback) in the multimodal condition could be accurately modelled as the integration of two feedback sources with the characteristics estimated in the conditions with AM and FM (Fig. 4). Moreover, the task performance (i.e., precision and accuracy of the matching task forces) was highest with multimodal feedback (Fig. 1).

Multimodal encoding was proposed by Valle and colleagues as a biomimetic approach (i.e., evokes parallel rate coding and recruitment)^9^. In agreement with that study, we found multimodal encoding to support closed-loop control better than AM or FM. In addition, our results suggest that the key aspect of multimodal encoding leading to superior control performance is that this approach enables sensor fusion by conveying two separate information streams through the same stimulation point. For natural sensory systems, the changes in rate coding and recruitment in a population of sensory nerves often do not lead to independent percepts but serve to provide one input to the central nervous system indicating, e.g., the magnitude of the stimuli in that sensory modality. For example, progressive activation of more muscle spindle receptors and their rate of action potential generation with an increase in muscle acceleration serves to provide a unified perception of muscle movement^14^. Conversely, the fact that FM and AM in electrotactile stimulation enable two largely independent perceptions (the quality of tapping/vibration and its intensity, respectively; see *Introduction*) appears to be the primary mechanism underlying the superior performance. However, methodological differences between our study and that of Valle and colleagues may limit the degree to which this conclusion also applies to the findings of that previous study. Specifically, Valle and colleagues used intraneural stimulation which evoked sensations in the phantom hand. In addition, this stimulation method can elicit a diverse set of sensations, some of which may have a more continuous nature^10^ compared to non-invasive stimulation that evokes mainly a sensation of tapping/vibration^18^. Moreover, a larger range of stimulation frequencies (up to 1 kHz as opposed to up to 65 Hz in this study) was used^9^. At high stimulation frequencies, individual stimulation pulses become indistinguishable, and FM may effectively feel like AM^19^. Finally, Valle and colleagues used non-linear encoding, which may better emphasize the task-relevant information. For example, non-linear encoding reflecting the force as well as the rate of change in force has been found to enable better performance for an object recognition task than linear force encoding^10^. Therefore, the approach applied in this study should be repeated using other stimulation methods before it can be claimed that benefits of multimodal encoding are always attributable to MLE-type integration of two distinct streams of information encoded in stimulation amplitude and frequency.

The results showed that when the CNS estimates the magnitude of force generated by the hand, it relies to a larger degree on the electrotactile feedback than on the natural force feedback (${W}_{electro}$> 0.5) irrespective of which modulation scheme is applied. As extensively discussed in a previous study^24^, this confirms the remarkable ability of the nervous system to adopt the sensation of tingling on the arm as the primary source of information regarding grasp force magnitude after just a few minutes of training. With multimodal encoding, the relative weight was > 0.9 in half of the subjects, indicating an almost complete reliance on electrotactile feedback. Moreover, the results showed that the estimated weights predicted functional outcomes, as the target forces were most accurately matched in the multimodal condition (Fig. 1). On a more general level, these findings support the notion that the experimental approach used in this study can be used as an objective measure for comparison of different feedback encoding strategies. Currently, feedback systems are typically evaluated based on their performance in a functional task performed with and without feedback^6^. The usability of the supplementary feedback, however, may be masked by cross-study differences in prosthesis type, user experience, task constraints, and the degree to which other task-relevant sources of feedback is available^6^. Possibly, as a results of such differences, inconsistent outcomes have been reported^6^, which indicates the need for more objective and generalizable evaluation criteria. To compensate for this, recent studies have proposed a battery of tests with varying requirements for task speed and accuracy, as well as for user attention and the degree to which visual feedback provided task-relevant information^25,26^. However, a simpler approach may be to estimate and compare relative weights in the sensory integration process as in this study, since it eliminates many of the potentially confounding factors and quantifies exclusively the ability of the CNS to interpret the feedback.

The finding that the nervous system appears to identify electrotactile multimodal modulation of a feedback signal in one channel as two independent signals may have other relevant implications. Specifically, this suggests that two different signals can be encoded in the same stimulation pattern (one in amplitude and another in frequency) and still be perceived accurately. Simultaneous encoding of many variables, such as interaction force across multiple locations of the prosthetic hand and kinematic variables (e.g., hand aperture), is relevant for human–machine interfaces, including prosthetic control^27,28^. It has been shown that electrotactile and vibrotactile stimulation can be perceived independently when placed on the same skin location^29^, but the ability to convey more variables via one stimulation channel may reduce cost, power consumption, and need for maintenance. Previously, Mayer and colleagues investigated this strategy in a simplified way, by asking subjects to identify one of three constant carrier frequencies (100, 400, or 750 Hz) during amplitude modulated vibrotactile feedback reflecting prosthesis grasp force^21^. The ability to convey two independent continuous signals, however, would greatly increase the bandwidth of the transmitted information. Whether this is indeed possible using concurrent modulation of frequency and amplitude as well as the required cognitive demands to decode such multivariable feedback, however, needs to be investigated in future studies. Moreover, the results of this study show that this potential benefit (larger bandwidth) comes with the cost of a reduction in perception accuracy (Fig. 1B). If the feasibility of the multivariable feedback is indeed confirmed, multimodal modulation would therefore allow selecting the trade-off between accuracy (single variable) and bandwidth (two variables) to best accommodate the requirements of a specific application.

In addition to the able-bodied participants, the study also included a transradial amputee as a representative of the potential target population. This subject participated in one session with multimodal modulation to provide a preliminary assessment of whether the sensory integration of the electrotactile feedback is altered when the electrodes are placed on the stump as opposed to an intact forearm. The test revealed that the sensation threshold, JND and the electrotactile weight (${W}_{electro}=0.84$) were similar to those obtained in able-bodied subjects. These findings are consistent with previous studies that did not identify differences in the touch-pressure sensibility in the stump and intact arms of adults with acquired upper extremity amputations^30,31^. Although verification in a larger population of amputee subjects is needed, this finding suggests that the main conclusions of the study also apply to prosthesis users.

In conclusion, supplementary electrotactile feedback with multimodal encoding enabled superior force matching ability compared to AM and FM. The results indicated that this was related to the fact that multimodal encoding is processed by the CNS as two independent signals, thereby increasing the reliability of the feedback signal according to MLE. The findings have implications for our basic understanding of how the brain processes artificially evoked sensory inputs, and for the design of bidirectional human–machine interfaces, such as for prosthetics. Specifically, for electrotactile stimulation a multimodal encoding strategy will optimally enable the user to perceive and exploit the feedback.

## Methods

### Participants

Fourteen able-bodied subjects (7 males and 7 females, 23.37 ± 7.13 years) and one left-hand transradial amputee (female; 49 years; the amputation occurred in 2009 following a traumatic accident) were recruited. The experiment was conducted in accordance with the declaration of Helsinki and approved by the ethics committee of Region Nordjylland, Denmark (reference number N-20190036). The subjects signed an informed consent form before commencing with the experiment.

### Experimental setup

**Figure 5:**
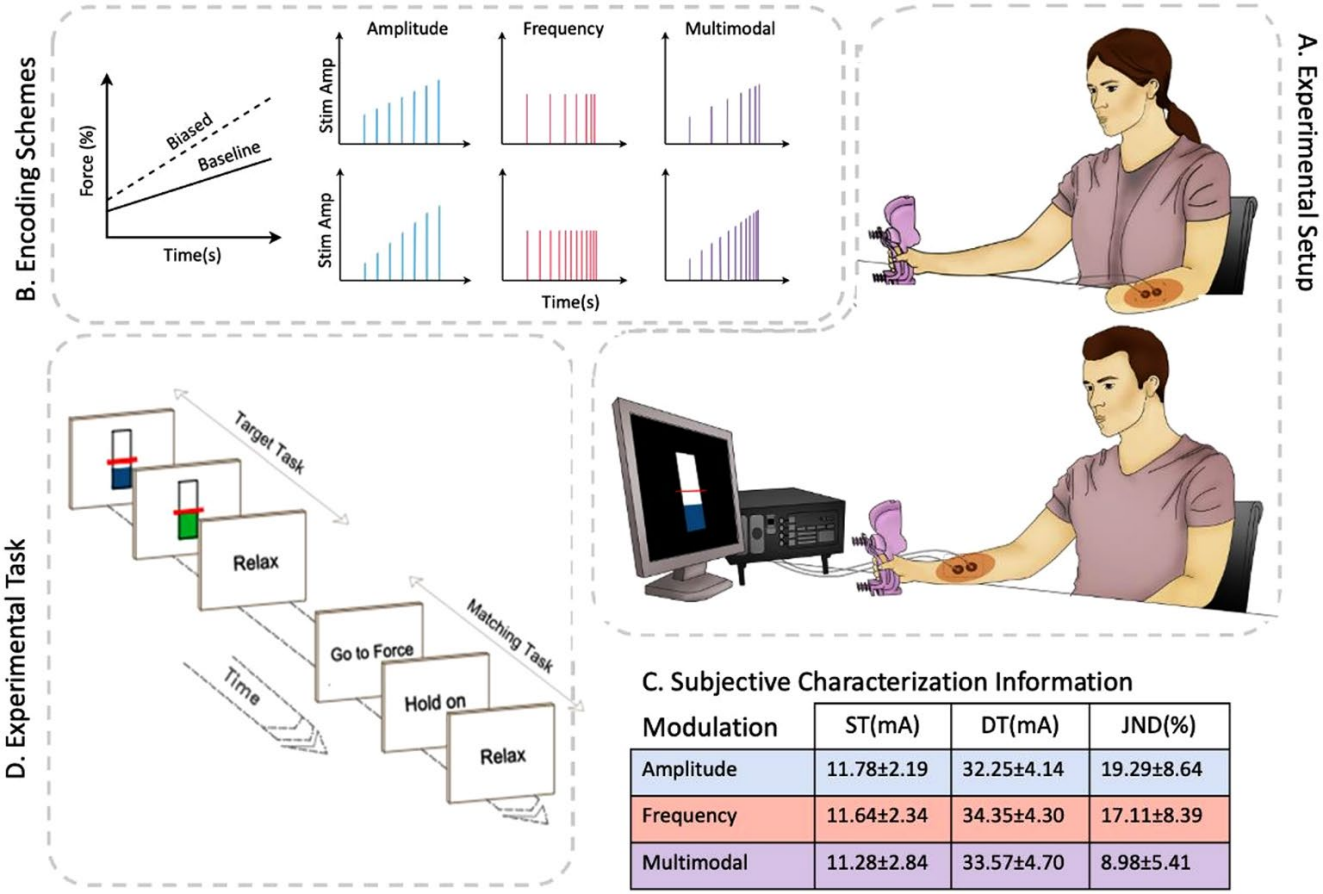
The experimental setup (**A**) included a grip force dynamometer, a standard PC, an electrical stimulator connected to a pair of stimulation electrodes located on the lower arm of the able-bodied subjects and on the lateral region of the amputee’s stump. Both able-bodied participants and the amputee participant felt the stimulation as localized sensations under the electrodes (highlighted in red). The recorded force signal was sampled and processed on a PC, where it was linearly encoded into pulse frequency and/or pulse amplitude (**B**) generated by the stimulator. In addition, in some conditions a visual representation of the generated force was shown on the computer screen by a floating bar. In this way, the subjects received streams of information regarding the generated grip force from up to three different sensory modalities: through vision, natural force feedback and electrotactile stimulation. Before the experiment began, the electrical stimulation was calibrated based on subjective characterization information (**C**). In the primary experimental task (**D**), each trial consisted of two contractions. First, the subject was asked to generate the specific force by reaching the target force (random value in the range 15–30%MVC, illustrated by a red horizontal line on the screen) and to hold this force as accurately as possible over a period of 5 s (*target subtask*). After relaxing for at least 5 s, the subject was asked to generate the same force but without visual feedback and to hold it for 3 s (*matching subtask*). In the *biased* condition the mapping between the force and the stimulation parameters (intensity, frequency, and both) was manipulated (**B**, bottom panel) so that the parameter rate of change was higher compared to the baseline condition (**B**, top panel). This was done for each of the three encoding schemes (AM, FM, multimodal) on three separate days.

The experimental setup and task were similar to that used in a previous study^24^. During the experiment, the subject was asked to hold and squeeze a grip force dynamometer (G200, Biometrics Ltd, USA) with their dominant hand (the amputee subject used her intact hand). In the able-bodied subjects, a pair of stimulation electrodes (Dura-Stick Self-Adhesive Premium Stimulating Electrodes, round, Ø: 3.2 cm) were placed on the radial side of the forearm, at one third of the length distally from the elbow and for the amputee subject, the electrodes were placed on the lateral proximal region of her stump (Fig. 5A). In all subjects, the stimulation evoked a localized sensation below the electrode without radiating or phantom sensations. The grip force signal was sampled and encoded in the electrotactile stimulation in real time using different encoding schemes, as explained in *Experimental Procedure*. All electrical stimuli consisted of trains of square-wave and biphasic pulses (pulse-width: 50 µs) generated by a stimulator (DS8R Biphasic Constant Current Stimulator, Digitimer, USA). In addition, in some parts of the experiments the generated force was presented on a computer screen, where a vertical bar indicated the instantaneous force.

### Experimental procedure

The experiment consisted of three sessions conducted in three consecutive days. In each session, the force matching task was performed with either AM, FM, or multimodal encoding (random order). Before the force matching task of each session, the subject went through a brief training session to learn the mapping between grasp force and the sensation evoked by the electrotactile stimulation. Moreover, the evoked sensations were evaluated psychometrically. While the able-bodied subjects participated in all three sessions, the amputee subject took part only in one session (multimodal encoding). In each session, the subject sat comfortably in front of a desk. In the beginning of each session, the MVC force was measured by asking the subject to grasp the hand dynamometer with maximum strength for 5 s. The mean force during the force plateau was extracted. The average force across the three repetitions (separated by > 1 min) was adopted as the MVC. During the experiment, the force was expressed in a normalized scale (0–1), corresponding to 0–30% MVC range. Next, the stimulation amplitude required for detection (sensation; ST) and discomfort (DT) were determined for 1-s bursts of stimulation at 42.5 Hz using the method of limits^32^ (Fig. 5C). This frequency value was the baseline frequency for the estimation of JND and the carrier frequency in amplitude modulation encoding (see details below). Furthermore, for all three modulation schemes, the JND was estimated using the staircase procedure (see *Just noticeable difference* section for details).

Next, the electrical stimulation parameters were adjusted according to the psychometric tests (Fig. 5C), and the closed-loop stimulation system was introduced to the subject. In this system, the instantaneous force signal (*f*) was encoded in stimulation pulse amplitude (amplitude modulation; AM), pulse frequency (frequency modulation; FM) or combination of AM and FM (multimodal modulation). In each experimental session one of these encoding schemes was applied in random order. In AM, the amplitude of the stimulation pulses (*A*) was proportional to grasping force according to the following linear relationship:

1 $$A\left(t\right)=B\times [0.8\cdot (DT-ST)\cdot f\left(t\right)+ST]$$

where ST and DT indicate the sensation and discomfort thresholds, respectively, and B is a multiplication factor that depends on the condition of the trial (*see below*). Prior to the experiment, we ensured that stimulation amplitudes would not exceed the discomfort threshold during the biased trials by reducing the value assigned to DT in Eq. (1), if necessary. For one subject with a high JND (37%) and DT at 39 mA, DT was scaled by 0.85 to ensure that the stimulation current at the highest target force did not exceed 37 mA. For all other subjects, this scaling was not necessary. In FM, the frequency of the stimulation pulses (*F*) was proportional to grasping force in the range 5–65 Hz, as indicated by Eq. (2):

2 $$F\left(t\right)=B \times [60\cdot f\left(t\right)+5]$$

The stimulation amplitude in FM was set to halfway between ST and DT to ensure a clear, comfortable sensation (pulse width: 50 µs). The carrier frequency in AM was set to 42.5 Hz.

Finally, the multimodal encoding condition was obtained by simultaneously modulating frequency and amplitude according to Eqs. (1) and (2). Importantly, this implementation of the three encoding schemes ensured that, on average, the net charge delivered to the subject was similar across all schemes.

The relations between force and stimulation parameters described by Eqs. (1) and (2) could be altered by changing the value of the parameter *B*. As indicated by Eqs. (3), *B* was assigned the value of 1 in the baseline settings. However, in some trials, the relations were biased by a value different from 1, thereby introducing a mismatch between the grip force and the stimulation with respect to the baseline condition (Fig. 5B). Importantly, this mismatch was unnoticeable by the subject since it was set to 80% of the magnitude of JND expressed in percent of amplitude (in Eq. 1), frequency (in Eq. 2), or both in the multimodal case.

3 $$B=\left\{\begin{array}{c}1, in\,Baseline\\ (1+\frac{0.8\cdot JND}{100}), in\,Bias\end{array}\right.$$

The training was conducted at the beginning of each session using the chosen modulation scheme (AM, FM or multimodal). The participants underwent two training runs to learn the baseline mapping between the stimulation and grasping force. Throughout the training runs, therefore, the baseline setting (*B* = 1; Eq. 3) was applied. Each run consisted of three blocks of approximately 2 min. In each block, in 12 consecutive trials, the subject had 6 s to match the target force and 4 s to relax before the next trial started. In the first run, the subject was asked to generate a specific force level randomly selected in the range 15–30% MVC. The instantaneous force was indicated visually by a target line on the screen. The next training run was similar to the first (in duration and number of contractions), except that the visual feedback was absent for the first three seconds, provoking the subject to rely only on natural and electrotactile feedback to generate the target force. The bar reappeared during the last three seconds of the trial, allowing the subject to correct the matching force if necessary.

As in our previous study^24^, in the primary experimental task, the subject was first asked to generate a target force (15–30% MVC) for 5 s, while receiving information about the generated force via visual, natural and electrotactile feedback. In this “target” subtask, the electrotactile feedback used the baseline mapping (Eq. 3), and hence, the relation between grip force and stimulation was similar to that learned in the training. After a 5-s pause, the visual feedback was removed, and the subject was asked to recreate the target force by relying only on natural feedback and the sensation evoked by electrotactile stimulation (“matching” subtask). The subjects performed 5 blocks with 12 trials per block (total of 60 trials) of the force matching task, comprising the target and matching subtask, in each of the three sessions. In half of the trials per block, the biased mapping (Eq. 3) was applied. The order of the baseline and biased trials was randomized. The subject was not informed that the mapping was biased in some trials. The experimenter paid special attention to whether the subject noticed the bias during the experiment and took note of any indications of this. Furthermore, after the finalization of the third session the subject was asked explicitly whether he/she noticed the bias.

### Just noticeable difference

The subject’s JND was assessed for FM, AM and multimodal modulation. Specifically, the subject was asked to discriminate the perceived intensity of pairs of stimulation pulse trains that varied in amplitude, frequency, or both. In this context, the JND was defined as the smallest change in the stimulation parameter expected to yield 75% correct discrimination. To minimize the duration of the experiment, this JND value was estimated using the adaptive weighted staircase procedure with 2 alternative forced choices^33^ in each of the three modulation conditions as in our previous study^22^. The step sizes of the weighted staircase (step down being three times smaller than the step up) were adopted to estimate the JND at 75% correct discrimination. A reference and a test stimulus (train of stimulation pulses lasting 1 s each) were delivered to the subject in random order separated by an interval of 0.5 s without stimulation. For each trial, the subject was asked to indicate if the first or the second pulse train had the highest value of the tested parameter (frequency, amplitude or both). Following the correct answer, the difference between test and reference stimulus (amplitude/frequency) was decreased for the next trial. If the answer was incorrect, the test stimulus was increased. If the subject did not guess correctly after a trial of successful detection, or vice versa, this was defined as a *reversal*. The staircase procedure was repeated until 7 reversals were recorded. The JND was computed as the difference between the average value of the stimulation parameter (frequency/amplitude) at the reversal points and the reference value and was expressed as a percentage of the reference value.

In AM, the amplitude of the reference stimulus was set halfway between ST and DT (reference amplitude) while the first test stimulus was set at an amplitude 30% higher than the reference. The stimulation frequency was 42.5 Hz (reference frequency). The step sizes were 0.2 mA and 0.6 mA following a correct or an incorrect answer, respectively (i.e., weighted staircase approach). To reduce the number of trials, the step size prior to the first wrong answer was set to 0.6 mA. A similar procedure was used for FM (reference frequency: 42.5 Hz). In the multimodal modulation condition, the test and reference stimuli values from AM and FM were adopted. Moreover, the step size from AM was used, which in turn constrained the frequency step size to maintain the linear relation between these two parameters. Consequently, the frequency step size depended on ST and DT for each subject. Finally, to ensure the same resolution of JND across the multimodal and FM conditions, the step size for the FM condition was determined in the same way. Across subjects, the average frequency step size following a correct answer was 0.74 ± 0.10 Hz (range: 0.60–0.93 Hz).

### Subjective evaluation

The subjective quality of the sensation elicited by the electrotactile stimulation was evaluated by a short questionnaire after each session. The subjects were asked to rate the pleasantness and efficiency (i.e., the degree to which the subjects perceived the feedback as useful during the task) of the stimulation. A numerical rating scale from 0 to 10 was used to assess comfort and efficiency in which 0 represented “very uncomfortable” or “non-efficient” and 10 represented “very comfortable” or “very efficient”.

### Data analysis

First, the average force from the plateau phase (*f*_*mt*_) of the matching subtask (3–6 s after the onset of the matching subtask when the hold on message appeared on the screen; Fig. 5D) was calculated for each trial. Prior to further analysis, the outliers in *f*_*mt*_ were removed from the dataset. The criterion for outliers was *f*_*mt*_ > 10% MVC from the target force or trials with coefficient of variation (standard deviation/mean of *f*_*mt*_) > 20%. On average, 0.29 ± 0.65 trials were excluded out of the 60 trials per session for each subject, equivalent to 0.5 ± 1.1% of all trials. We suspected that such trials reflected that the subject temporarily lost focus and failed to remember the target force during the matching task.

The *f*_*mt*_ was then expressed as a percentage of the target force, and the average ${f}_{mt}$ for each of the two conditions (*baseline* and *biased*) and three encoding schemes (AM, FM, and multimodal) as well as their variance (${\sigma }_{AM}^{2}$, ${\sigma }_{FM}^{2}$ and ${\sigma }_{multi}^{2}$) across trials were computed. We refer to the absolute error between the average *f*_*mt*_ and the target (i.e., 100%) as the matching subtask error.

Exploiting the MLE-framework, the relative weight assigned by the central nervous system to the electrotactile feedback (${W}_{electro}$) in the estimation of the grip force was computed for the three encoding conditions (AM, FM, multimodal) by the following equation, as in^24^:

4 $${W}_{electro}=\frac{{f}_{mt}\left\{bias\right\}-{f}_{mt}\{baseline\}}{{f}_{electro}\left\{bias\right\}-100}=1-{W}_{natural}$$

where ${f}_{mt}\{bias\}$ and ${f}_{mt}\{baseline\}$ is the average matching subtask plateau force in biased and baseline trials, respectively. ${f}_{electro}\{biased\}$ refers to the force that the subject should have generated in order to receive the same level of electrotactile stimulation (i.e., the same frequency and/or amplitude) as when producing the target force in the target task. *W*_*electro*_ therefore indicated how much the subjects responded to the bias in the electrotactile feedback for each encoding scheme, i.e., if they “followed” the biased electrotactile cue or ignored the cue and relied on the natural feedback. In the next step of the analysis, we addressed the hypothesis that the performance in the experimental task with multimodal modulation could be predicted from the performance in the AM and FM conditions based on the MLE-framework. In^24^, it was shown that the grip force produced by the subject could be estimated by the weighted average of the state information obtained from the electrotactile and natural feedback, respectively, as indicated by Eq. (5):

5 $${S}_{final}= {W}_{electro}{S}_{electro}+{W}_{natural}{S}_{natural}$$

where S indicates the state estimates (i.e., the instantaneous grip force) from the sensory sources (electro and natural) as well as the final estimate that determines the force produced during the task. The weights (*W*) in Eq. (5) were associated with the reliability (variance) of the input as follows:

6 $${W}_{electro}=\frac{{\sigma }_{natural}^{2}}{{\sigma }_{natural}^{2}+{\sigma }_{electro}^{2}}$$

and

7 $${W}_{natural}=\frac{{\sigma }_{electro}^{2}}{{\sigma }_{natural}^{2}+{\sigma }_{electro}^{2}}$$

By extension, the hypothesis implies that in the multimodal condition, the final estimate is the integration of the states indicated by AM and FM as two separate inputs, as well as of natural input, thereby extending Eq. (5) as follows:

8 $${S}_{final}= {W}_{electro[AM]}{S}_{electro[AM]}+{W}_{electro[FM]}{S}_{electro[FM]}+{W}_{natural}{S}_{natural}$$

To test this hypothesis, we investigated if the variance of *f*_*mt*_ and relative weight of the electrotactile feedback in the multimodal condition could be predicted from the variance and weights measured in the AM and FM conditions. To this end, we first estimated the variance of each individual feedback source (i.e.,${\sigma }_{natural}^{2}$ and ${\sigma }_{electro}^{2}$) from the data collected in the experimental sessions with AM and FM. The variance of ${f}_{mt}$ that was measured in the AM and FM sessions can be described as:

9 $${\sigma }_{electro+natural}^{2}=\frac{{\sigma }_{electro}^{2}{\sigma }_{natural}^{2}}{{\sigma }_{electro}^{2}+{\sigma }_{natural}^{2}}$$

By combining and rearranging Eqs. (6) and (9) as well as (7) and (9) we find that:

10 $${\sigma }_{electro}^{2}=\frac{{\sigma }_{electro+natural}^{2}}{{W}_{electro} }$$

11 $${\sigma }_{natural}^{2}=\frac{{\sigma }_{electro+natural}^{2}}{{W}_{natural} }$$

In this way, Eqs. (10) and (11) enabled the estimation of the variance of each of the two feedback sources in the AM and FM conditions for each subject, since ${\sigma }_{electro+natural}^{2}$ was equivalent to the variance of the generated forces *f*_*mt*_ (i.e., ${\sigma }_{AM}^{2}$, ${\sigma }_{FM}^{2}$) while the relative weights were calculated using Eq. (4).

Next, the estimated values of ${\sigma }_{electro}^{2}$ and ${\sigma }_{natural}^{2}$ was used to predict the variance of ${f}_{mt}$ with multimodal modulation (${\widetilde{\sigma }}_{multi}^{2}$). Here, the value of ${\sigma }_{natural}^{2}$ was set to the average of the two values, ${\widetilde{\sigma }}_{natural[AM]}^{2}$ and ${\widetilde{\sigma }}_{natural[FM]}^{2}$, obtained from the AM and FM sessions, respectively. The predicted variance (${\widetilde{\sigma }}_{multi}^{2}$) could then be compared to experimentally measured variance from each subject in the multimodal condition. Specifically, the predicted variance was estimated according to Eq. (12):

12 $${\widetilde{\sigma }}_{multi}^{2}=\frac{1}{\frac{1}{{\widetilde{\sigma }}_{natural}^{2}}+\frac{1}{{\widetilde{\sigma }}_{electro[AM]}^{2}}+\frac{1}{{\widetilde{\sigma }}_{electro[FM]}^{2}}}$$

In addition, the predicted weight assigned to electrotactile feedback in multimodal condition (${\widetilde{W}}_{electro[multi]})$ was then determined using Eq. (6). In this case, ${\sigma }_{electro}^{2}$ was estimated by combining the estimated values of ${\sigma }_{electro}^{2}$ from the AM and FM conditions (as in Eq. 9):

13 $${\sigma }_{electro[multi]}^{2}= \frac{{\widetilde{\sigma }}_{electro[AM] }^{2}{\widetilde{\sigma }}_{electro[FM]}^{2}}{{\widetilde{\sigma }}_{electro[AM]}^{2}+{\widetilde{\sigma }}_{electro[FM]}^{2}}$$

Linear regression analysis was applied to investigate the correlation between the predicted (${\widetilde{\sigma }}_{multi}^{2}$) and observed variance (${\sigma }_{multi}^{2})$ as well as the predicted and observed weight of the multimodal modulation across all subjects.

The statistical analysis was performed using R Statistical Software (version 3.0.2; R Foundation for Statistical Computing, Vienna, Austria). The outcome measures including JND, error, variance and ${W}_{electro}$ were compared using a repeated measure ANOVA with one within-subject factor (type of modulation) and one between- subject factor (the order of modulation). Kolmogorov–Smirnov test was used to check the normality of data, Mauchly’s test for data sphericity and a post hoc Bonferroni correction was applied to perform pair-wise comparisons. Friedman test and Wilcoxon rank sum test were used to compare the subjective evaluation scores. For all tests, the significance level was set at $p$ < 0.05.

## Data Availability

The datasets generated during and/or analyzed during the current study are available from the corresponding author on reasonable request.
